# Grazing Induced Shifts in Phytoplankton Cell Size Explain the Community Response to Nutrient Supply

**DOI:** 10.3390/microorganisms9122440

**Published:** 2021-11-26

**Authors:** Evangelia Charalampous, Birte Matthiessen, Ulrich Sommer

**Affiliations:** GEOMAR, Helmholz Center for Ocean Research, 24148 Kiel, Germany; bmatthiessen@geomar.de (B.M.); usommer@geomar.de (U.S.)

**Keywords:** phytoplankton, cell size, copepod grazing, nutrients

## Abstract

Phytoplankton cell size is important for a multitude of functional traits such as growth rates, storage capabilities, and resistance to grazing. Because these response traits are correlated, selective effects on mean community cell size of one environmental factor should impact the ability of phytoplankton to cope with other factors. Here, we experimentally apply expectations on the functional importance of phytoplankton cell size to the community level. We used a natural marine plankton community, and first altered the community’s cell size structure by exposing it to six different grazer densities. The size-shifted communities were then treated with a saturated nutrient pulse to test how the changes in community size structure influenced the mean community growth rate in the short-term (day 1–3) and nutrient storage capacity in the postbloom phase. Copepod grazing reduced the medium-sized phytoplankton and increased the share of the smallest (<10 µm^3^) and the largest (>100,000 µm^3^). Communities composed of on average small cells grew faster in response to the nutrient pulse, and thus confirmed the previously suggested growth advantage of small cells for the community level. In contrast, larger phytoplankton showed better storage capabilities, reflected in a slower post-bloom decline of communities that were on average composed of larger cells. Our findings underline that the easily measurable mean cell size of a taxonomically complex phytoplankton community can be used as an indicator trait to predict phytoplankton responses to sequential environmental changes.

## 1. Introduction

Phytoplankton community structure is currently changing due to anthropogenic global change with consequences for the structure and functioning of the associated pelagic food webs [1,2,3,4]. Trait-based approaches are believed to be a key tool to understand the causes and consequences of phytoplankton change. While they simplify the vast taxonomic complexity, trait-based approaches also provide a way to explain phytoplankton community property changes because phytoplankton traits are functionally relevant. As a way of complexity reduction Litchman and Klausmeier [5] proposed the identification of a few “master” traits based on correlations among traits. Among these, cell size is a strong candidate that can be easily measured. Linear measures of cell size in phytoplankton spans four to five orders of magnitude; cell diameters range from less than a micrometer up to several millimeters [6]. Size affects several other traits that are either related to nutrients and growth, or to loss factors [5,7]. Across studies, cell size correlates negatively with growth rate [6,8,9,10,11,12] and the ability to take up nutrients at low concentrations [13,14]. It correlates positively with the abilities to rapidly take-up replete nutrients provided in a pulsed way, and to store nutrients [12,13,15,16], which means that slower growing larger cells can preserve the gained biomass for longer compared to smaller cells. In particular, the relationship between cell size and growth-related traits was extensively examined using culture experiments. The only exception from the otherwise consistently shown negative relationship between maximal (nutrient saturated) growth rate (μ_max_) and cell size [6,8,9,10,11,12] was postulated by Marañon et al. [12]. By including for the first time pico- and small nanoplankton Marañon et al. [12] experimentally showed a unimodal µ_max_–cell size relationship with a peak at ca. <100 μm^3^.

Grazing, besides of sinking, is one of the most important loss factors in phytoplankton. Grazing is, however, not only responsible for biomass reduction per se but also for mean community size shifts since specific grazers feed on specific size classes. Generally, consumer and prey sizes are positively correlated in aquatic systems [16,17]. Mesozooplanktonic copepods are among those grazers that strongly impact phytoplankton size distribution [16] both by direct size selective grazing (direct path) and/or indirect suppression of micrograzers (indirect path) [18,19,20]. Many phytoplankton species, however, developed morphological and physiological mechanisms to defend themselves against grazing or other loss factors, such as sinking. Among these is colony formation to increase their size, flagella that enable movement either to escape or to remain in the water column, or spine formation that slows down the sinking velocity and causes handling difficulties to potential grazers.

In nature, most environmental ”stressors” act simultaneously on communities, driving their response to either synergistic or antagonistic effects. Great difficulty is thus created in disentangling the influence of each one of the environmental factors on the community response. A first step to disentangle the individual effects of stressors is the use of situations, where the relative importance of stressors varies in time. In such situations, it can be seen how dominance of one stressor shapes the distribution of community traits (including size) and how this change in trait distribution might affect the community level ability to cope with a stressor gaining later dominance. Vinebrooke et al. [21] concept of sequential stressors carried this scenario to the extreme to facilitate experimental and modeling analysis of double stressor effects. In this context, it is critical to understand the links among functional response and effect traits [22,23,24]. Even though Vinebrooke’s [21] concept refers to species diversity, we extend it to trait-based compositional changes, since environment-induced change of diversity can also be translated to a change in trait-diversity, which likely will further affect community performance under yet another environmental factor.

While there is ample knowledge from pure cultures about the size dependence of nutrient related traits, here we set out to test whether environmentally driven mean cell size changes can explain community performances in response to a second environmental driver. We designed a mesocosm experiment where a natural protist plankton community (autotrophic phytoplankton and heterotrophic protists) from the Baltic Sea was first subject to mesozooplankton grazing to manipulate size structure. Thereafter, we studied the response of the resulting communities to subsequent nutrient addition and depletion. We hypothesized, that the response should be primarily mediated by mean community cell size. Zooplankton grazing for size selection and nutrient limitation as subsequent factor was chosen because of its importance in the natural environment and because of its well-known effect on trait diversity.

Specifically, we expected that (i) grazing vulnerability of species is affected by cell size. In consequence copepod grazing will shift protist size distributions to a dominance of very small and very big cells. We further expected that (ii) communities dominated by small-celled individuals will respond faster to nutrient addition, since it is predicted that small sized species will show higher growth rates than bigger ones when nutrients will be available; and (iii) communities dominated by big cells will continue growing for longer under prolonged nutrient shortage, because of better storage capacity of big cells.

## 2. Materials and Methods

### 2.1. Experimental Design

An indoor mesocosm experiment with natural Baltic Sea plankton communities was conducted in temperature and light controlled climate rooms from 23 February until the 11 March 2016. Prior to the experimental onset (22 February) eighteen mesocosms of 250 L each were filled with seawater containing the plankton community from the Kiel Bight (Baltic Sea). The mesocosms were randomly distributed across three temperature and light controlled climate rooms each equipped with three LED light system units [25,26] and set in pairs under one light system unit. Daily irradiance patterns stayed constant over the course of the experiment and were computer controlled (GHL, Prometheus). The light−dark cycle was 11 h 50 min:12 h 10 min including dusk and dawn. Light supply and day length were aligned to the seasonal light patterns calculated by the astronomic model of Brock [27] for an approximated cloudless 21 March reduced to 60% of solar irradiance to account for underwater light attenuation.

Mesocosms were filled with a peristaltic pump taking water from 2 m depth in the Kiel Fjord (54°19′47.0″ N, 10°08′59.6″ E) and distributed equally trough a hose system into each of the mesocosms in the three climate rooms. According to previous experiments with the same system [25,26,28,29], unicellular plankton were well represented in in situ conditions, while copepods were heavily underrepresented.

Water temperature at the day of the filling was 4 °C (in situ) and was gradually shifted to 10 °C to provoke growth in the plankton communities and to mimic the mean water temperature expected for March, in agreement with the programmed light conditions. For this purpose, room temperature was set at 7 °C on the 22 February and at 10 °C on the 23. The mesocosm water reached the desired temperature on the 25 February. The experiment started after the water in the mesocosms reached the desired temperature. During the temperature acclimation, fluorescence in the mesocosms increased from 0.18 (value the day after the filling) to 0.28 (value at the experimental start.

To minimize sedimentation and to achieve a good distribution of the plankton communities the mesocosms were gently stirred manually using a paddle every 12 h.

To address our scientific questions, the experiment consisted of two subsequent phases. The first one (i) included the size shift of the plankton communities caused by zooplankton grazing (grazing phase). The second one (ii) included the community response to nutrients (nutrient phase).

**Grazing phase**—the mesocosms were treated with a triplicated gradient of 6 different copepod (*Acartia tonsa*) densities (0, 10, 20, 40, 80, 160 ind L^−1^). The copepods originated from cultures and net catches (54°20′32.7″ N 9°58′05.7″ E) and were acclimated to the experimental temperature at the same time as the water in the mesocosms. The 0 ind L^−1^ treatment level did not involve additional grazers to serve as ambient treatment control. The mesocosms were exposed to grazing for 7 days starting the day the mesocosms’ water reached the desired temperature (25 February).

After the seventh day of the grazing phase the mesocosms were sampled and the water microscopically examined regarding the predicted size responses of the grazing treatments. Accordingly, the two mesocosms showing the maximum difference in community size structure and an intermediate one, were chosen to provide the experimental source communities for the nutrient phase. The selected mesocosms belonged to 0 ind L^−1^, the 40 ind L^−1^ and of the 160 ind L^−1^ pregrazed treatments. In the following text, the new treatments will be referred to as 0 ind L^−1^, 40 ind L^−1^, and 160 ind L^−1^ pregrazed treatments depending on the grazing pressure they had experienced.

**Nutrient Phase**—18 smaller (30 L) mesocosms were filled with water from the three designated pregrazed (0, 40 and 160 ind L^−1^) source communities (6 of each pregrazed treatment) after the removal of the grazers. Grazers were removed by sieving the community through a 200 μm mesh size plankton net. The new mesocosms were filled gradually to achieve a good distribution of the plankton communities. Two different nutrient treatments, nutrient-enriched (N) and control (C), were applied to each of the three pregrazed communities and replicated three-fold which resulted in six different treatment combinations of pregrazed and nutrient-treated communities.

The target concentration of the nutrient enriched treatment was 16 µmol L^−1^ SiO_4_, 16 µmol L^−1^ NO_3_ and 1 µmol L^−1^ PO_4_, i.e., Redfield ratio (16 nitrogen/1 phosphorus). Nutrients were added according to the premeasured concentration at the end of the grazing phase in each of the three chosen pregrazed mesocosms. The control treatments (C) did not receive any additional nutrients.

### 2.2. Sampling and Measurements

Salinity, pH, temperature and relative fluorescence as a proxy for phytoplankton biomass were measured daily to monitor the experiment. The relative fluorescence was measured using a fluorometer 10-AU (Turner Design, CA, USA) immediately after the sampling. Sampling was performed directly after mixing and water was syphoned with a tube to a 5 L container. From this, subsamples were taken for phytoplankton analyses (phytoplankton counts, cell measurements and flow cytometry) and dissolved inorganic nutrient measurements. The sampling dates were the beginning of the experiment, the end of the grazing phase/start of nutrient phase, then the 3rd and 10th day of the nutrient experiment to test the short (3 days) and midterm response (10 days) of the communities to nutrients.

Water for dissolved nutrient analyses were filtered through pre-washed (10% HCl) cellulose acetate filters and stored in −20 °C until analyzed following the protocols of Hansen & Koroleff [30]. Samples for microscopic phytoplankton analysis were collected in brown bottles (200 mL) and fixed with Lugol’s iodine [31].

Phytoplankton (>5 μm) was counted according to the Utermöhl [32] method with an inverted microscope. In every sample at least 400 individuals were counted and at least 100 of the most abundant taxa. Directly after the sampling phytoplankton smaller than 5 μm was counted with a flow cytometer (FACScalibur, Becton Dickinson, Franklin Lakes, NJ, USA). Cell size measurements (linear dimensions) were made with the use of the AxioVision program (Zeiss, Oberkochen, Germany) and converted into cell volumes according to the geometric models of Hillebrand et al. [33]. Species biomass was calculated from the species total abundance multiplied with the individual cell volume. To test the suitability of a size based “classification” instead of a taxonomic characterization of phytoplankton, similar sized species were grouped in six size classes. Specifically, the size classes were picoplankton (<10 μm^3^), small (10–100 μm^3^), medium (100–1000 μm^3^), big (1000–10,000 μm^3^), very big (10,000–100,000 μm^3^), and extremely big (>100,000 μm^3^) sized species. Size classes biomass were determined based on taxa cell volume.

Vulnerability of species and size classes to grazing was indicated by the regression slopes of the abundance of each species and size class in response to grazer density. Positive values indicate that a species was not grazed, and negative values indicate that a species was among the preferred as food source. The steeper the slope the stronger is the susceptibility to grazing by the copepod *A. tonsa*.

Growth rates (μ) (for phytoplankton: total community, size classes and species; for heterotrophs: ciliates and heterotrophic dinoflagellates) were calculated for the short-term response (3 days) by the difference of the logarithmically transformed biomass (b_i_) between the three different sampling dates divided by the time (t_i_) difference: μ = (ln b_3_ − ln b_1_)/(t_3_ − t_1_).

Community mean cell volume (V_mean community_) was calculated based on the % contribution of each taxon to total abundance (p_i_) multiplied by the taxon biovolume (V_i_): V_mean community_ = Σp_i_ × V_i_

The ability of the communities to preserve the gained biomass under extended nutrient shortage (Biomass preservation) was calculated as the difference between the absolute value of the increment of the biomass at the end of the experiment (B_10_) and the biomass build up each community achieved after the addition of the nutrients (B_3_) (nutrient phase, day 3): Biomass preservation = B_10_−B_3_.

To compare our results with that of the literature (see discussion), we recalculated the growth rate-cell size relationship according to a double log_10_-transformation. In addition, the new relationships were corrected with Eppley’s [34] Q10-value of 1.88 to account the difference in growth between 10 and 20 °C.

### 2.3. Statistical Analysis

The effects of grazing intensity (independent variable) on total community biomass and abundance, as well as size class and species abundance were analyzed through Generalized Linear Models (GLMs) (family Gamma, link = log), which allowed us to deal with heteroscedasticity in the data. The relationship between cell size and species or size class grazer vulnerability was described using a second-degree polynomial regression model (function LM). ANOSIM was used to test if the pregrazed communities were significantly different in size class contribution to total biomass. ANOSIM was conducted twice, before (biomass and abundance based) and after (biomass based) sieving for copepods. After sieving, Simper analysis was used to test which size class shifts were responsible for the differentiation of the treatments at the onset of the nutrient phase. Effects of pregrazing and nutrient availability on biomass increase during the first three days of the nutrient phase are described qualitatively. Effects of pregrazing and nutrient availability on growth rate of the differently sized plankton communities in the nutrient phase were tested using two-factorial ANOVA. One factorial ANOVA was used to assess the effect of pregrazing to particulate organic nutrients ratios (carbon/nitrogen, carbon/phosphorus, nitrogen/phosphorus) at the end of the experiment. The effect of cell size to the growth rate of the nutrient added communities, the biomass loss from day 3 to 10 and the particulate organic nutrients ratios (carbon/nitrogen, carbon/phosphorus, nitrogen/phosphorus) was analyzed through a simple linear regression model (LM). Logarithmic transformation was used for the carbon/nitrogen, carbon/phosphorus, nitrogen/phosphorus ratios to satisfy the statistic assumptions. All statistics were performed using R version 3.4.1 (30 June 2017).

## 3. Results

### 3.1. Grazing Phase

**Figure 1:**
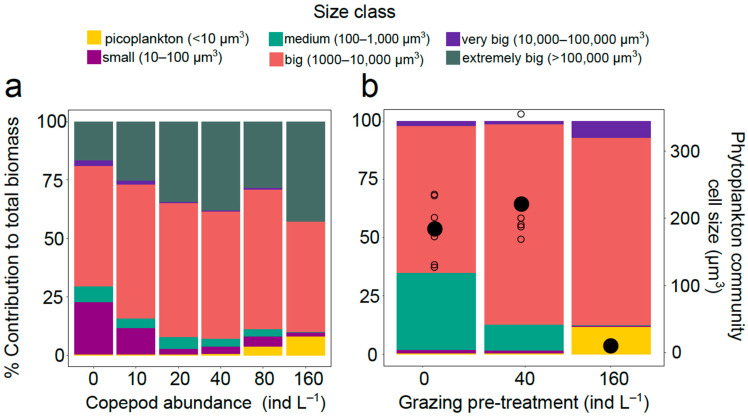
% Contribution to total biomass of size classes (picoplankton, small, medium, big, very big, extremely big) (**a**) in differently pregrazed treatments after seven days of grazing, (**b**) in selected pregrazed treatments after sieving for copepods. Panel (**b**) shows, also, mean community cell size (µm^3^) (secondary axis) in grazing pretreatments (0, 40 and 160 ind L^–1^) after sieving for copepods. Empty circles indicate mean community cell size of replicates and black circles indicate mean cell size of each pregrazed treatment.

**Effect of grazing on community cell size structure.** Phytoplankton started to grow immediately in response to the light supply and temperature increase in the climate chambers (Figure S1a in Supporting Information). While grazer density did not impact total phytoplankton biomass (Figure S2a), it affected total abundance (positively) (Figure S2b) and the community size structure (Figure 1a) (ANOSIM: R = 0.2296, *p* = 0.023 *** for biomass-based and R = 0.1942, *p* = 0.029 *** for cell abundance-based calculation). By reducing the intermediate phytoplankton size classes (small (10–100 μm^3^), medium (100–1000 μm^3^) and very big (10,000–100,000 μm^3^) cells) (Figure 1a and Figure S3b,c,e) copepod grazing increased both the share of the picoplankton (<10 µm^3^), and the extremely big (>100,000 µm^3^) size classes (Figure 1a and Figure S3f). Only the big size class (1000–10,000 μm^3^) was not affected by grazer density (Figure S3d). However, the change in community size structure was not translated into a clear change of the mean community cell size, because both extremes profited from grazing.

Most species were affected by grazing in the same way as their respective size class (compare Figures S3 and S4). The same was the case for species belonging to size classes that were not affected by grazing. For example, the species *Chaetoceros danicus* and *Chaetoceros* sp., which were the most abundant species of the size class big cells (1000–10,000 μm^3^; >90% contribution to size class biomass in all treatments), showed no response to grazing and neither did the size class (Figures S3 and S4).

**Figure 2:**
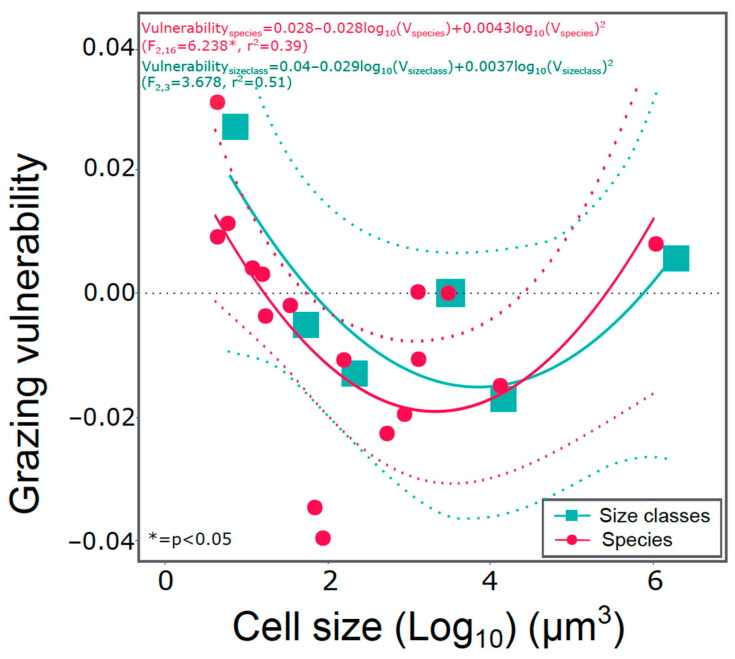
Grazing vulnerability as a function of mean cell size of size classes (green color data) and of different species (red color data). Grazing vulnerability calculated as regression slope of cell abundance in response to copepod density (see also Figures S3 and S4 in Supporting Information). Dashed lines represent 95% confidence intervals.

The size structure differences between the three pregrazed communities chosen as treatments for the subsequent nutrient phase of the experiment (i.e., after removing copepods with the sieve) were even more pronounced (Figure 2b) (ANOSIM: R = 0.67, *p =* 0.001 ***, biomass-based). While they did not contain extremely big cells (removed by sieving procedure), the distinctive altered size distribution towards more picoplankton and very big cells was kept (Figure 1b). On average, community mean cell size was smallest with highest grazing intensity (Figure 1b). Detailed analyses of the size classes responsible for the pregrazed communities’ discrimination are shown in Table S1.

### 3.2. Response to Nutrients

**Biomass**—nutrient enrichment (N) increased phytoplankton biomass (Figure S1b,c in Supporting Information). During the first three days of the nutrient phase, among the nutrient enriched treatments, the most intense (160 ind L^−1^) pregrazed treatment showed the highest absolute increase in growth (Figure S1c). In the nutrient controls (C), only the 0 ind L^−1^ pregrazed treatment showed a small biomass increase (Figure S1d) until day three, although the available nutrients were depleted almost to the same level as in the more intense pregrazed nutrient controls (C) (Figure S5).

From day 3 until day 10, biomass decreased in all nutrient treatments (N and C) (Figure S1c,d). Among the 3 nutrient enriched treatments (N), the 160 ind L^−1^ pregrazed ones showed the strongest/most pronounced decline, while the 0 ind L^−1^ and 40 ind L^−1^ pregrazed communities managed to better preserve the biomass built-up until day 3 (Figure S1c). Among the nutrient control treatments (C) the 0 ind L^−1^ pregrazed lost most biomass from day 3 until day 10 (Figure S1d).

**Growth rate**—nutrient addition increased the growth rates of all size classes and hence mean community growth rate (Table 1) (Figure S6). At the same time, in both nutrient-enriched (N) and nutrient control (C) treatments, pregrazing decreased the growth rates of picoplankton (<10 μm^3^) and large cells (>10,000 μm^3^) cells (µ_max_ at 0 ind L^−1^ pregrazed treatments) (Table 1) (Figure S6), and increased the growth rate of small cells (10–100 μm^3^) (µ_max_ at 160 ind L^−1^ pregrazed treatments) (Table 1) (Figure S6). The interactive effect of both grazing and nutrients was neither significant for the total community nor for any size class response (Table 1). For effects of nutrient enrichment and grazing on the single species, please see Table S2 and Figure S7.

Growth rates of heterotrophic protists during the first three days varied widely between positive and negative values and were mainly attributed to the growth of heterotrophic dinoflagellates and less on the growth of ciliates. Heterotrophic dinoflagellates showed positive growth in both 0 ind L^−1^ pregrazed nutrient treatments (N and C) and in the nutrient controls (C) of the 40 ind L^−1^ pregrazed treatments (Figure S8).

**Nutrient analysis**—except for NH_4_, the available dissolved nutrients in the nutrient enriched communities (N) were almost exhausted already from the third day of the nutrient phase in all treatment combinations (Figure S5 in Supporting Information). NH_4_ increased in all treatments until day 10 of the nutrient phase to not more than 2 µmol L^−1^. All nutrient enriched treatments (N) were in the transition between nitrogen and phosphorus limitation at day 10, reflected in the nitrogen/phosphorus ratio in the particulate matter of 16.7 ± SD1.52. Both carbon/nitrogen (ANOVA: F_2,6_ = 14.14 *) and carbon/phosphorus (ANOVA: F_2,6_ = 13.07 *) ratios showed the highest value (22.3 ± SD 0.34 and 388.1 ± SD 33.4 accordingly) at the 0 ind L^−1^ pregrazed treatments and the lowest at the 160 ind L^−1^ pregrazed (15.03 ± SD2.4 and 248.3 ± SD38.68 accordingly).

### 3.3. Cell Size Effect on Phytoplankton Traits

**Cell size effects on grazing vulnerability**—the grazing vulnerability, indicated by the regression slopes of phytoplankton abundance responses of size classes and species to grazer density in Figures S3 and S4, showed U-shaped quadratic relationships with cell size (Figure 2).

As such, medium-sized cells showed the highest vulnerability to grazing (Figure 2).

**Figure 3:**
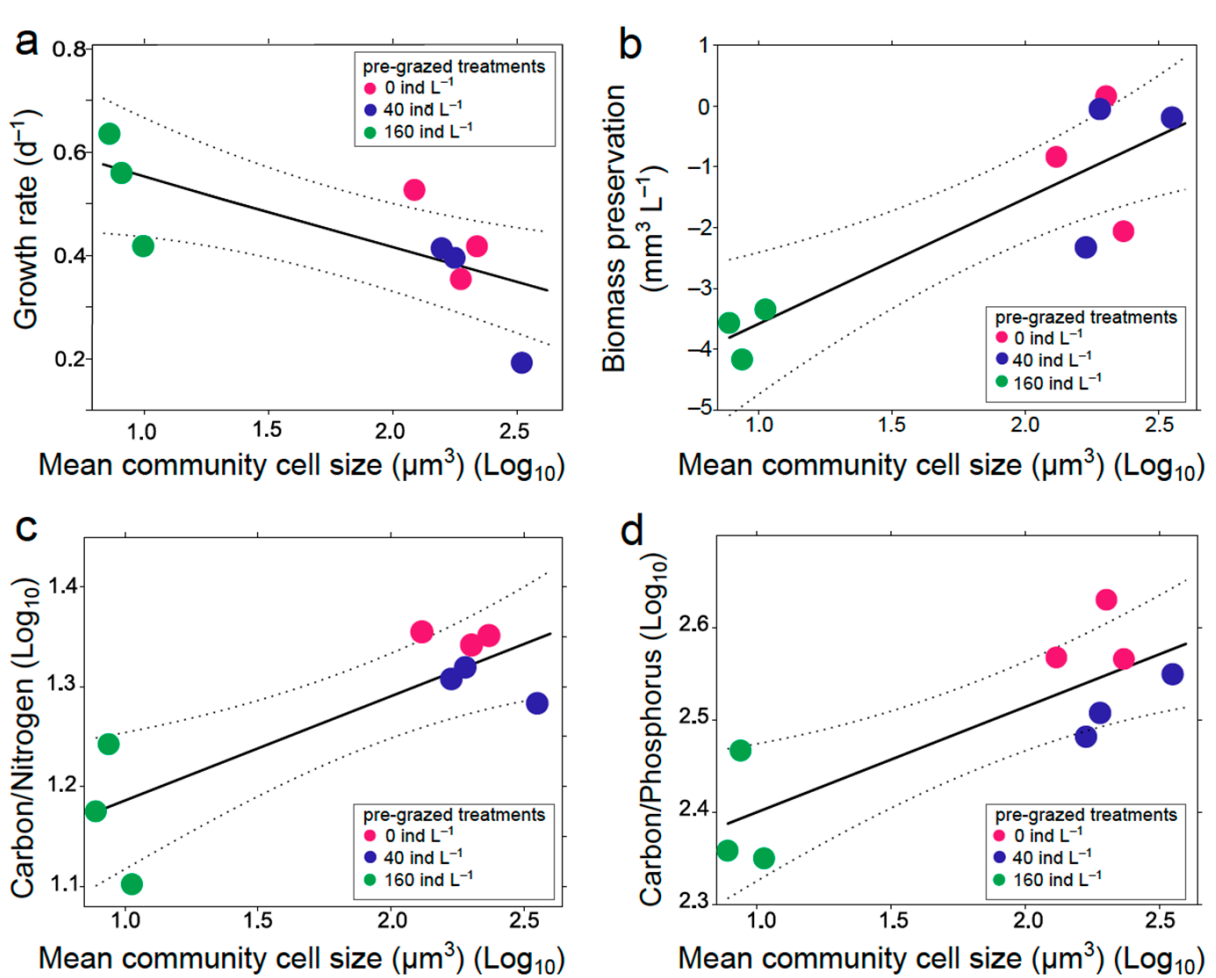
Regressions of (**a**) community growth rate (short-term response, experimental days 1–3), (**b**) postbloom biomass loss from day 3 to 10, (**c**) carbon/nitrogen, and (**d**) carbon/phosphorus ratios in nutrient treatments (N) at day 10 of nutrient phase in response to mean community cell size. Dashed lines show 95% confidence intervals.

**Growth rates**—mean community growth rates significantly declined with mean community cell size (Figure 3a). The relationship between community mean cell size (μm^3^) and community growth rate is described as:

μ_mean community size_ = 0.68 − 0.13 log_10_V (F_1,7_ = 7.5 *, r^2^ = 0.44)

* = *p* < 0.05

**Biomass preservation from day 3 to 10**—biomass preservation under extended nutrient shortage significantly increased with community mean cell size (Figure 3b) and is described as

Biomass preservation = −5.64 + 2.06 log_10_V (F_1,7_ = 19.59 *, r^2^ = 0.69)

* = *p* < 0.05

**Carbon/nitrogen and carbon/phosphorus ratios**—carbon/nitrogen (Figure 3c) and carbon/phosphorus (Figure 3d) significantly increased with mean community cell size and followed the equations

log_10_(carbon/nitrogen) = 1.08 + 0.1 log_10_ V (F_1,7_ = 15.03 *, r^2^ = 0.63) and

log_10_(carbon/phosphorus) = 2.28 + 0.11 log_10_ V (F_1,7_ = 14.94 *, r^2^ = 0.63),

* = *p* < 0.05

respectively.

## 4. Discussion

By exposing a natural community of unicellular plankton first to grazing and then to nutrient stress we were able to show that shifts in mean cell size can indeed be used as an indicator trait to predict the response of a natural community to sequential environmental alterations. Our mesocosm experiment is embedded in the discussion about the double nature of phytoplankton traits; firstly, as an indicator of a particular environmental stress and, secondly, as an attribute to cope with environmental stress.

**Response to grazing.** The present results corroborate the outcome of previous experiments with natural plankton communities showing that copepod grazing can affect community size structure [18,20], mainly benefiting the small and the very large phytoplankton taxa (expectation i). Our findings indicate a clear size shift in the communities described by the response of the size classes to grazing and the % contribution of size classes to total biomass. The benefit of small phytoplankton in response to mesozooplankton grazing can be explained partly by suppression of protozoan grazers but also by removal of their larger competitors. However, also very big or well defended cell share the exclusion from grazing such as *Coscinodiscus wailesii* and hard-shelled dinoflagellates [35,36]. Our findings agree with these results. By increasing copepod abundance, we achieved to suppress the abundance of all size classes except the picoplankton (<10 μm^3^) and the extremely big cells (>100,000 μm^3^). This resulting increase of the extreme size classes indicated that a classical calculation of mean community cell size did not reflect the size shift, although the use of abundance data in the calculation proved to be a better descriptor. The same response (lower or higher abundance) applied also to the corresponding individual taxa of the size classes. Taxa with cell size from around 100 μm^3^ to 1000 μm^3^ were the most vulnerable to grazing pressure, with two species *Cylindrotheca closterium* and *Skeletonema* sp. being the most vulnerable. On the contrary, the almost neutral (not favored or negatively affected) size class response of medium sized cells (100–1000 μm^3^), with *Cheatoceros* as the dominant species can be explained by the copepods not preferring them as food source. This “diet exclusion” of *Chaetoceros* species was also observed at the highly grazed treatments where most taxa were eaten and *Chaetoceros* was among the few if not the only abundant species that did not belong to picoplankton. We assume that their shape, especially the presence of long spines, was the determining factor for not being grazed.

**Figure 4:**
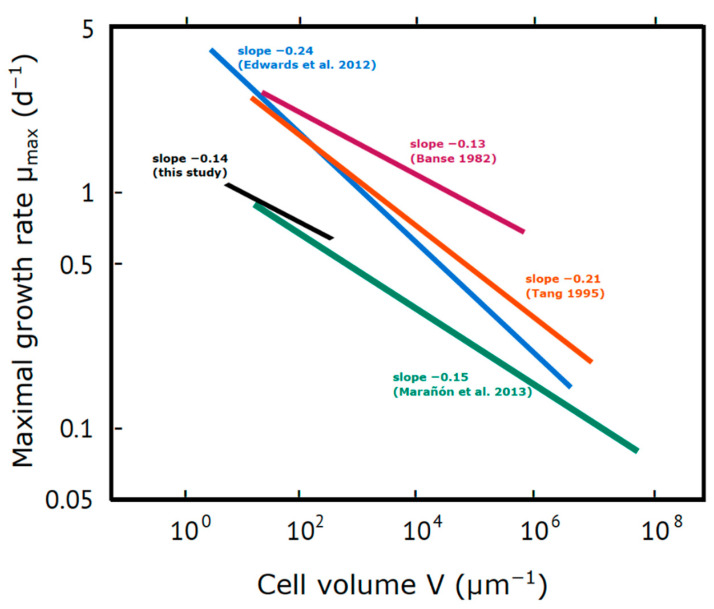
Graphical comparison of relationship between phytoplankton maximal growth rates and cell size resulting from this study (black line) and literature sources (measured at or recalculated for 18–20 °C).

**Response to nutrients**. The cell size effect on growth rates revealed a clear negative relationship between increasing cell size and growth rates (expectation ii). The comparable relationship describing the growth rate–cell volume relationship of our experiment with existing literature were:

for total community growth: μ = −0.2 V^−0.14^ (F_1,7_ = 5.48, *p* = 0.05, r^2^ = 0.36)

which is close to the −0.15-slope described by Marañón [12] for species of a volume larger than 40 μm^3^ (Figure 4). The slope of the relationship is much weaker than the one described by Edwards et al. [11] for marine species, but within the range of some earlier studies [8,10,37] (Figure 4). This weak relationship, compared to that of Edwards et al. [11], could be partly explained by the calculation method of the slope. We fitted our data on a linear model, whereas Edwards et al. [11] used a standardized major axis estimation which produces steeper slopes. In addition, the observed growth rates in our study were not necessarily the μ_max_ of each community or maybe not even close to it. The time frame of 3 days given to the communities to grow after nutrient addition was probably too big to reliably estimate µ_max_ because the communities possibly reached stationary growth at an earlier point. Moreover, the species involved in the equation consist of only diatoms and one flagellate (Cryptophyte: *Teleaulax* sp.). Dinoflagellates which in general have lower growth rates than diatoms [11,12,16,38,39] were not available for inclusion.

The timing of our sampling schedule was not optimal for testing expectation (iii) (bigger cells will better cope with prolonged nutrient shortage). Based on the temporal dynamics of previous experiments in the same experimental systems, we expected that until day 3 small phytoplankton would display their faster growth, while thereafter the larger phytoplankton would surpass them because of their longer maintenance of nutrient saturated growth. Unfortunately, bloom dynamics were faster than expected and all types of phytoplankton had reached their peak already at day 3 (or possibly shortly afterwards, but clearly before day 10). Nevertheless, the outcome of the experiment revealed some interesting size related trends. The communities with bigger mean cell size managed to have a pronounced weaker biomass loss after day 3 than the communities dominated by smaller cells. However, the smaller cell sized communities show a lower carbon/nitrogen and carbon/phosphorus ratio at day 10. This could mean that smaller cells might preserve a healthier state since they can continue their ribosomal metabolic activities, produce proteins, and continue growing for longer. Smaller cells, because of their low surface to volume ratios might not have the same storage capacities as the bigger cells but they showed a better ability to respond to a nutrient spike and also better nutrient utilization ability in low nutrient concentrations, as shown already during the first three days of the experiment. The slower decline of the larger phytoplankton should, therefore, be attributed to a higher resistance to the prevalent loss factors, in our case most probably grazing by mesozooplankton.

**The role of history**. The application of the initial stressor altered the response to nutrient conditions in the differently sized communities. The results are consistent with previous findings by our group [38], in which light intensity instead of grazing was manipulated. The mean community size negatively correlated with growth rates of the new communities, in agreement with the results described in Charalampous et al. [38], indicating that the suppression of individuals of specific size by the first stressors lead to a remaining community of species responding differently to the second stressor. However, effects had different direction for initial growth rates after nutrient addition and carbon/nitrogen, carbon/phosphorus (positive effects of smaller cell sizes) and the ability to sustain the gained biomass both in the present experiment and in Charalampous et al. [38]. The combination of the knowledge retrieved from these relationships describe the advantages and disadvantages that occur in the communities depending on the initial direction of the trait shift. For example, if the trait distribution resulting from one stressor drives the community mean cell size to smaller values, then the communities will respond faster to nutrients (negative correlation with growth rate) and might have a healthier state in terms of metabolic activity (lower carbon/nitrogen and carbon/phosphorus ratios), but will not achieve the same nutrient conversion into biomass, as a bigger sized community would have, and also will not be able to preserve this biomass for long periods.

## 5. Conclusions

The question of the applicability of cell size as indicator trait on natural ecosystems and also the accuracy of the allometric relationships is still open. The results from this work confirm that cell size is potentially an applicable indicator trait of growth and loss factors of phytoplankton communities. Cell size was confirmed to be the determinant factor for species grazing vulnerability, although the shape of a species might alter the sensitivity to specific grazers. Picoplankton and very big cells profited from copepod grazing since copepods removed the medium sized algae. The extreme size difference of abundant species in a phytoplankton community caused by copepod grazing reveals a weakness of cell size as a metric to reveal the size shift of the community. When testing for the response of the different sized communities to nutrient stress a significant relationship between cell size and growth rates was retrieved. More precisely, the small sized communities showed a faster growth rate after the nutrient pulse anrbond achieved a higher biomass built up in short-term, however, they preserved the least biomass on a longer period. Our results also indicate the importance of the ecological history of phytoplankton communities, since trait shifts (here cell size shifts) induced by one environmental factor (grazing) impacted the ability of the community to respond to another environmental factor (nutrients).

## Figures and Tables

**Table 1:** ANOVA results showing short-term effects (days 1–3) of factors pregrazing, nutrient addition and interaction between them on mean community growth rate and growth rate of size classes. * = *p* < 0.05.

Growth Rate	Factor	Df	Sum Sq	Mean Sq	F
Total Community	Pregrazing	2	0.06	0.03	2.8
	Nutrients	1	0.49	0.49	43.3 *
	Pregrazing × Nutrients	2	0.04	0.02	1.6
Size classes(μm^3^)					
Picoplankton (<10)	Pregrazing	2	1.31	0.65	66.4 *
	Nutrients	1	0.18	0.18	18.5 *
	Pregrazing × Nutrients	2	0.01	0.01	0.6
Small (10–100)	Pregrazing	2	1.52	0.76	20.5 *
	Nutrients	1	0.78	0.78	21.1 *
	Pregrazing × Nutrients	2	0.13	0.07	1.8
Medium (100–1000)	Pregrazing	2	0.32	0.16	1.4
	Nutrients	1	0.72	0.72	6.6 *
	Pregrazing × Nutrients	2	0.30	0.15	1.4
Big (1000–10,000)	Pregrazing	2	0.09	0.05	1.8
	Nutrients	1	0.68	0.68	27.5 *
	Pregrazing × Nutrients	2	0.06	0.03	1.2
Very big (>10,000)	Pregrazing	2	3.25	1.63	19.8 *
	Nutrients	1	0.66	0.66	8 *
	Pregrazing × Nutrients	2	0.12	0.06	0.7

## Data Availability

The data presented in this study will be openly available in PANGAEA (http://www.pangaea.de/, accessed on 25 November 2021).

## Supplementary Materials

The following are available online at https://www.mdpi.com/article/10.3390/microorganisms9122440/s1, Figure S1: (a,b) Time course of fluorescence changes as a proxy of total phytoplankton biomass in the two experimental phases. (a) Grazing phase: different symbols indicate the different treatments. (b) Nutrient phase: different symbols indicate the different treatments. Filled symbols indicate the nutrient shifted treatments, empty symbols the control treatments. (c,d) Time course of total phytoplankton biomass during the nutrient phase in 0 (circles), 40 (triangles) and 160 (squares) ind ind L^−1^ pre-grazed treatments (c) with added nutrients (N) and (d) without nutrients (C). Small symbols indicate the biomass of the replicates; big symbols indicate the average biomass of the treatments and SE, Figure S2: Effect of grazing on total phytoplankton biomass (a) and abundance (b) (modeled with GLM, family GAMMA, link = log). The grey area shows the 95% C.I. *= *p* < 0.05, Figure S3: Effect of grazer density on the abundance of different size classes (<10, 10–100, 100–1000, 1000–10,000, 10,000–100,000, >100,000 μm^3^) (modeled with GLM, family GAMMA, link = log). Grey area shows 95% C.I. * = *p* < 0.05, Figure S4: Effect of grazing on the abundance of different phytoplankton species (modelled with GLM, family GAMMA, link = log). The grey area shows the 95% C.I. * = *p* < 0.05, Figure S5: PO_4_, NO_3_, NH_4_ and SiO_4_ concentration in 0, 40 and 160 ind L^−1^ pre-grazed treatments during nutrient phase. Lighter symbols indicate the biomass of the replicates; darker symbols indicate the average nutrient concentration of the treatments, Figure S6: Total phytoplankton community and size classes (picoplankton (<10 μm^3^), small (10–100 μm^3^), medium (100–1000 μm^3^), big (1000–10,000 μm^3^) and very big (>10,000 μm^3^) cells growth rate (short term, days 1–3) for the nutrient (N) and control (C) pre-grazed treatments (0, 40, 160 ind L^−1^). Small circles represent the response of the replicates and big ones the mean between the replicates, Figure S7: Species growth rate (short–term, days 13) for the nutrient (N) and control (C) pre–grazed treatments (0, 40, 160 ind L^−1^). Small circles represent the response of the replicates and big ones the mean between the replicates, Figure S8: Species growth rate (short–term, days 13) for the nutrient (N) and control (C) pre–grazed treatments (0, 40, 160 ind L^−1^). Small circles represent the response of the replicates and big ones the mean between the replicates, Table S1: Simper analysis results for the contribution of each size group to the differentiation of the pre-grazed communities, Table S2: ANOVA results showing the short-term effects (days 13) of the factors pre- grazing, nutrient addition and the interaction between them on species growth rate. * = *p* < 0.05.

## Aknowledgements

The authors want to thank the student helper I. Stoltenberg for assistance in samplings and sample preparation. We gratefully acknowledge T. Hansen for general technical assistance and flow cytometry sample processing and B. Gardeler and C. Meyer for processing nutrient data. The authors also are grateful to Maria Moustaka–Gouni for her help in distinguishing the species from the flow cytometer, and microscopy.
