# Supplementary material for: Grazing Induced Shifts in Phytoplankton Cell Size Explain the Community Response to Nutrient Supply
Source: Microorganisms. 2021 Nov 26;9(12):2440. doi: 10.3390/microorganisms9122440 (PMC8708950; doi:10.3390/microorganisms9122440)
Supplement: Supplementary file 1 [file microorganisms-09-02440-s001.zip › Figure S1.pdf]

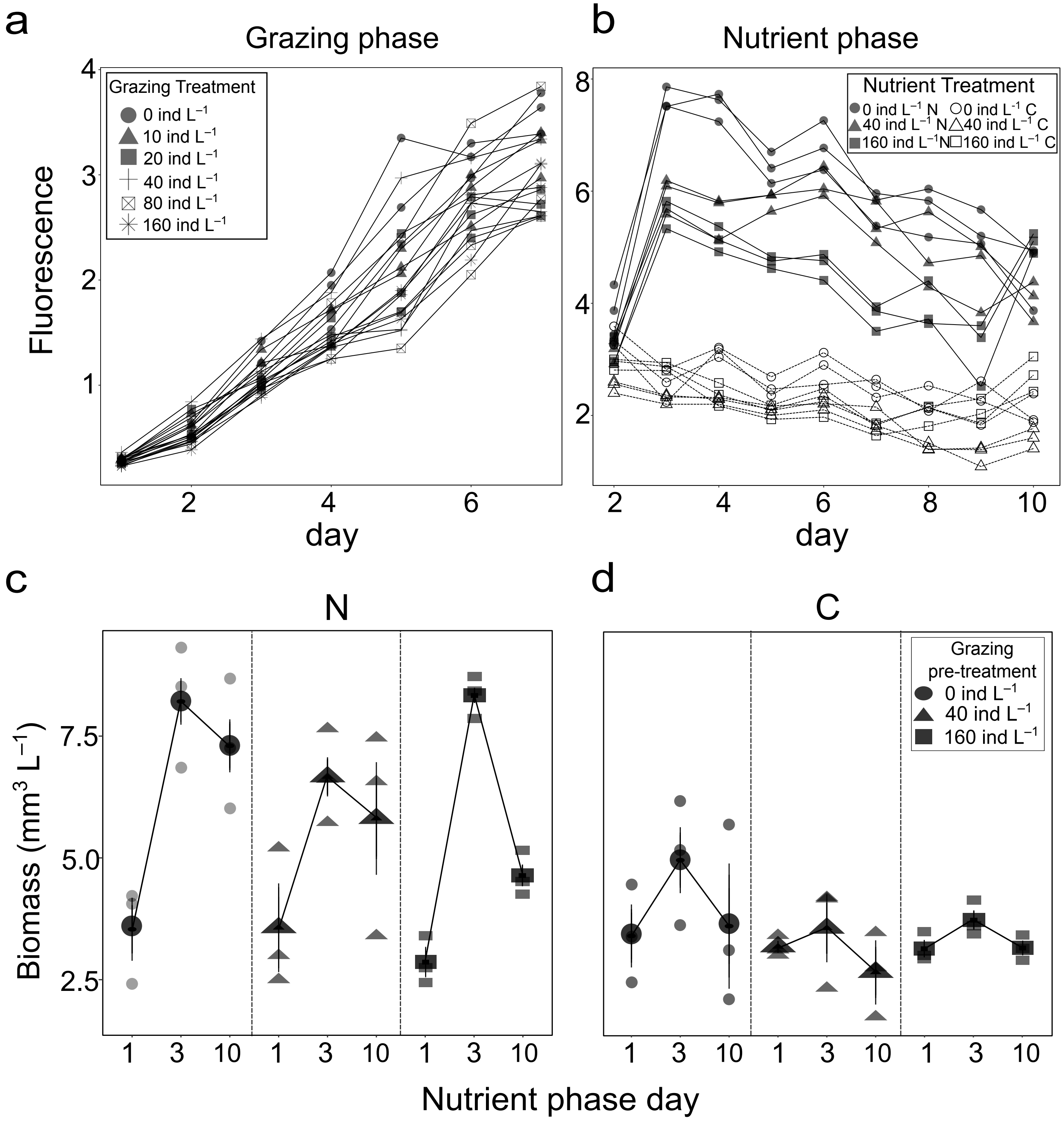

Figure S1: a–b)Time course of fluorescence changes as a proxy of total phytoplankton biomass in the two experimental phases. (a) Grazing phase: different symbols indicate the different treatments. (b) Nutrient phase: different symbols indicate the different treatments. Filled symbols indicate the nutrient shifted treatments, empty symbols the control treatments. c–d) Time course of total phytoplankton biomass during the nutrient phase in 0 (circles), 40 (triangles) and 160 (squares) ind L^{-1} pre-grazed treatments (c) with added nutrients (N) and (d) without nutrients (C). Small symbols indicate the biomass of the replicates; big symbols indicate the average biomass of the treatments and SE.
