# Supplementary material for: Grazing Induced Shifts in Phytoplankton Cell Size Explain the Community Response to Nutrient Supply
Source: Microorganisms. 2021 Nov 26;9(12):2440. doi: 10.3390/microorganisms9122440 (PMC8708950; doi:10.3390/microorganisms9122440)
Supplement: Supplementary file 1 [file microorganisms-09-02440-s001.zip › Figure S3.pdf]

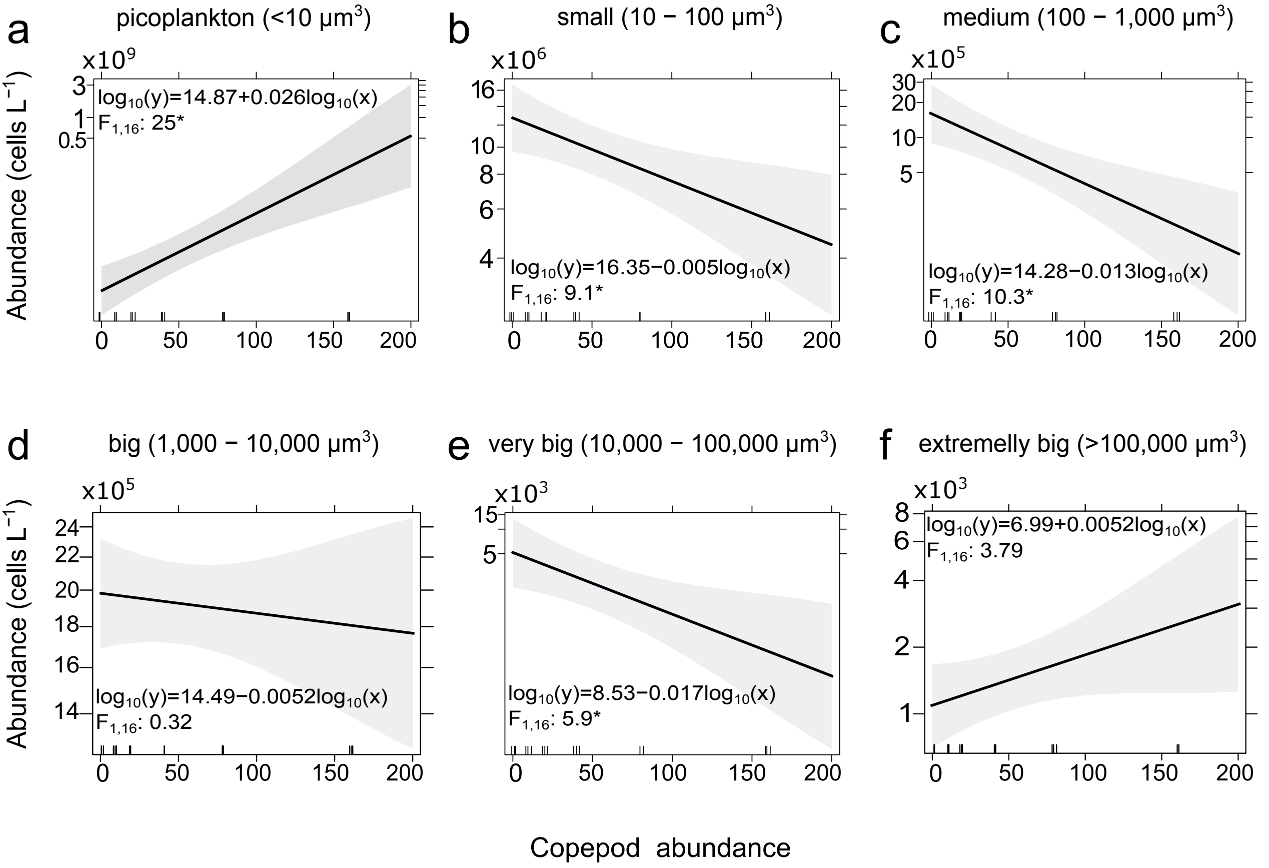

Figure S3. Effect of grazer density on the abundance of different size classes (<10, 10−100, 100−1,000, 1,000−10,000, 10,000−100,000, >100,000 μm^3) (modeled with GLM, family GAMMA, link=log). Grey area shows 95% C.I. *= $p<0.05$
