# Supplementary material for: Grazing Induced Shifts in Phytoplankton Cell Size Explain the Community Response to Nutrient Supply
Source: Microorganisms. 2021 Nov 26;9(12):2440. doi: 10.3390/microorganisms9122440 (PMC8708950; doi:10.3390/microorganisms9122440)
Supplement: Supplementary file 1 [file microorganisms-09-02440-s001.zip › Figure S4.pdf]

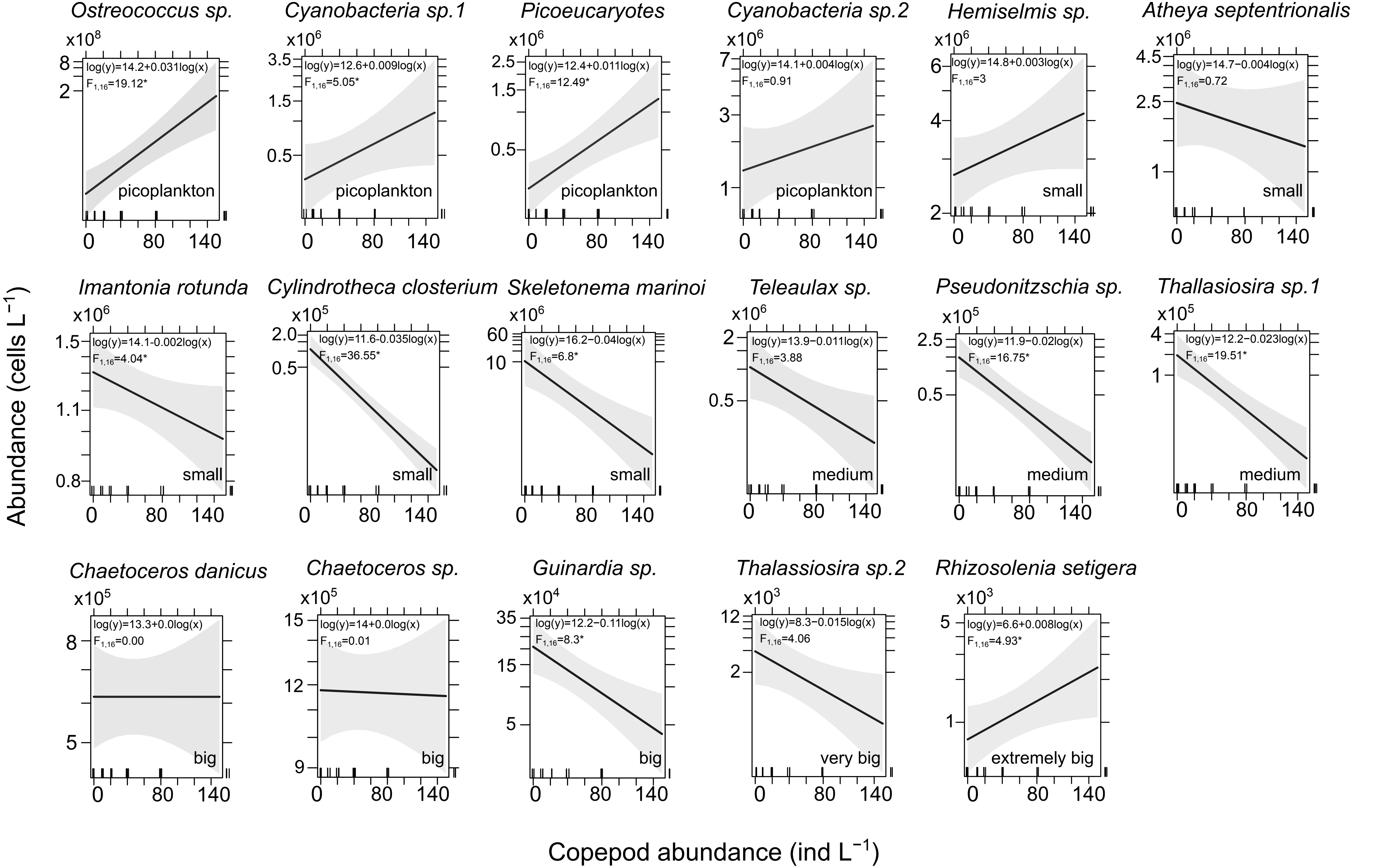

Figure S4. Effect of grazing on the abundance of different phytoplankton species (modelled with GLM, family GAMMA, link=log). The grey area shows the 95% C.I. $*=p<0.05$
