# Supplementary material for: Grazing Induced Shifts in Phytoplankton Cell Size Explain the Community Response to Nutrient Supply
Source: Microorganisms. 2021 Nov 26;9(12):2440. doi: 10.3390/microorganisms9122440 (PMC8708950; doi:10.3390/microorganisms9122440)
Supplement: Supplementary file 1 [file microorganisms-09-02440-s001.zip › Figure S5.pdf]

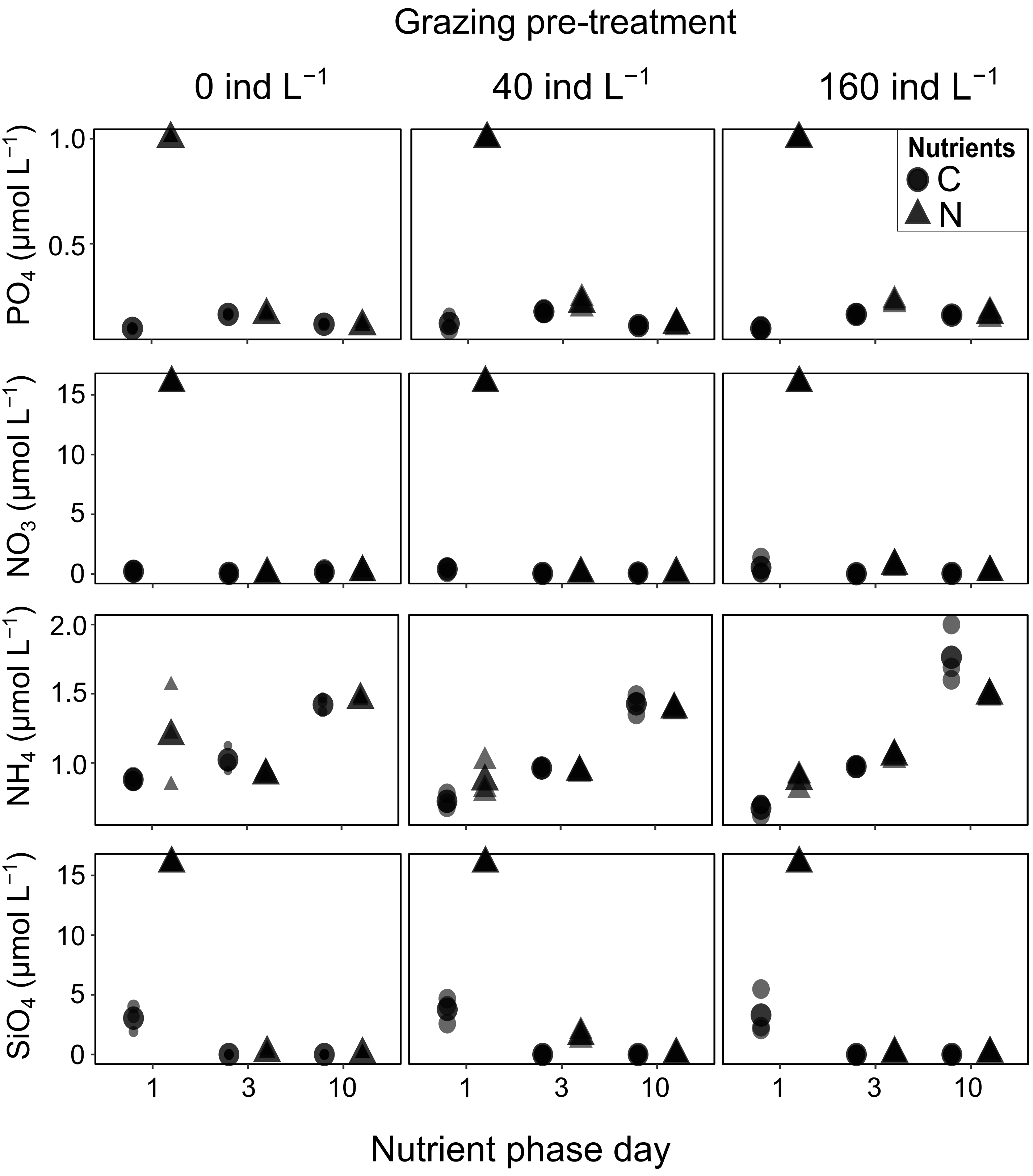

Figure S5. PO_4, NO_3, NH_4 and SiO_4 concentration in 0, 40 and 160 ind L^{-1} pre-grazed treatments during nutrient phase. Lighter symbols indicate the biomass of the replicates; darker symbols indicate the average nutrient concentration of the treatments.
