# Supplementary material for: Grazing Induced Shifts in Phytoplankton Cell Size Explain the Community Response to Nutrient Supply
Source: Microorganisms. 2021 Nov 26;9(12):2440. doi: 10.3390/microorganisms9122440 (PMC8708950; doi:10.3390/microorganisms9122440)
Supplement: Supplementary file 1 [file microorganisms-09-02440-s001.zip › Figure S7.pdf]

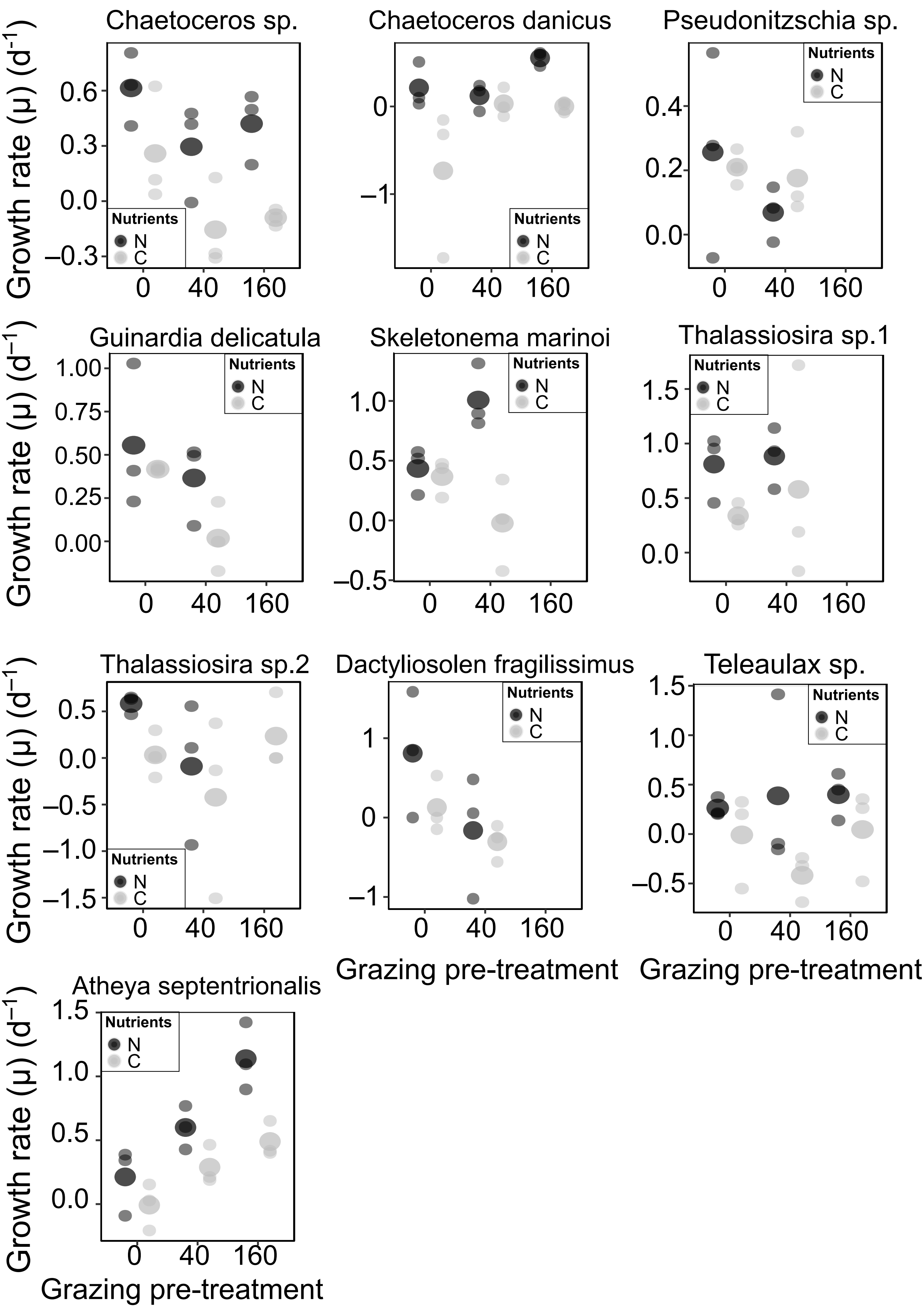

Figure S7. Species growth rate (short–term, days 13) for the nutrient (N) and control (C) pre–grazed treatments (0, 40, 160 ind L^{-1}). Small circles represent the response of the replicates and big ones the mean between the replicates.
