# Supplementary material for: Grazing Induced Shifts in Phytoplankton Cell Size Explain the Community Response to Nutrient Supply
Source: Microorganisms. 2021 Nov 26;9(12):2440. doi: 10.3390/microorganisms9122440 (PMC8708950; doi:10.3390/microorganisms9122440)
Supplement: Supplementary file 1 [file microorganisms-09-02440-s001.zip › Figure S8.pdf]

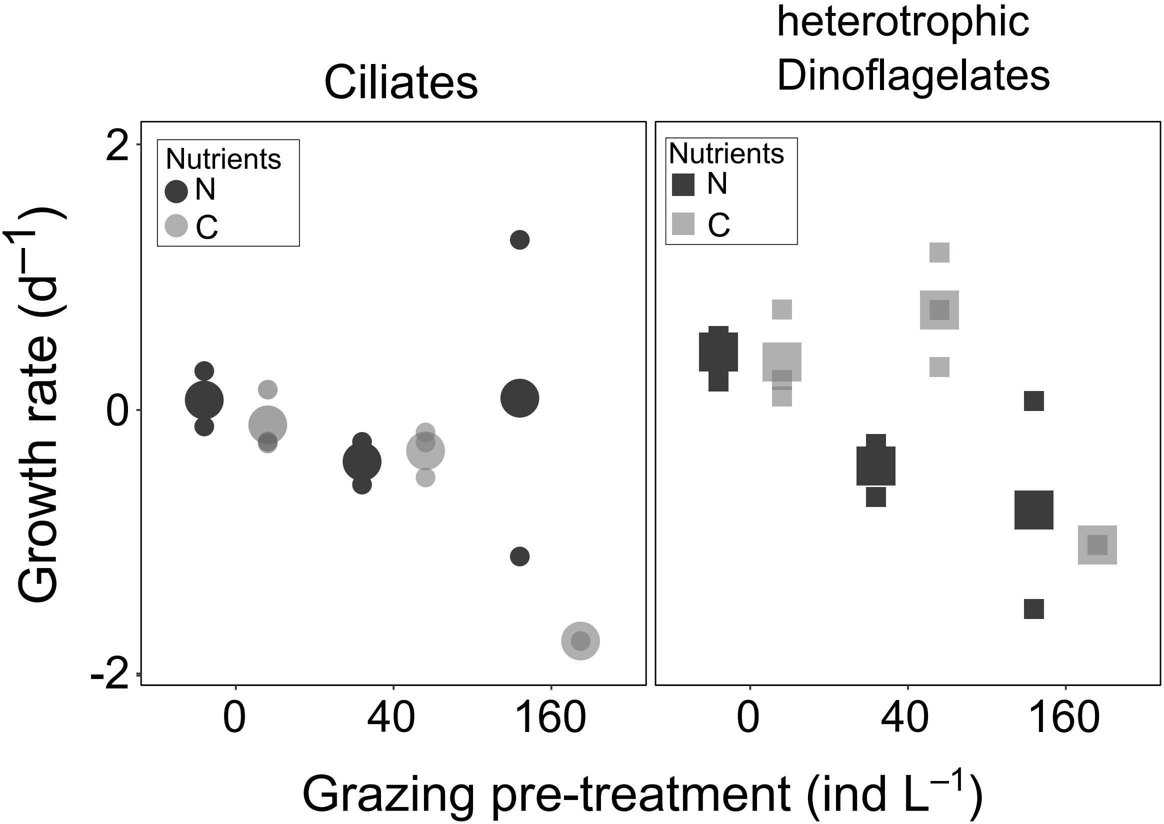

Figure S8. Short term (days 1–3) growth rate of Ciliates (circles) and heterotrophic Dinoflagellates (squares) for the nutrient enriched (N) and the control (C) treatments of the pre- grazed (0, 40 and 160 ind L^{-1}) treatments. Small circles and squares represent the response of the replicates and big ones the mean between the replicates.
