# Supplementary material for: Grazing Induced Shifts in Phytoplankton Cell Size Explain the Community Response to Nutrient Supply
Source: Microorganisms. 2021 Nov 26;9(12):2440. doi: 10.3390/microorganisms9122440 (PMC8708950; doi:10.3390/microorganisms9122440)
Supplement: Supplementary file 1 [file microorganisms-09-02440-s001.zip › Tables S1 and S2.pdf]

**Supporting Information**

**Table S1**: Simper analysis results for the contribution of each size group to the differentiation of the pre-grazed communities.

| Contrast between 0 – 40 ind $L^{-1}$ pre-grazed communities | |
|---|---|
| Size class ($\mu m^3$) | % |
| big (1,000–10,000) | 56.75 |
| medium (100–1,000) | 40.57 |
| very big (>10,000) | 1.69 |
| small (10–100) | 0.68 |
| picoplankton (<10) | 0.31 |
| Contrast between 0 – 160 ind $L^{-1}$ pre-grazed communities | |
| Size class ($\mu m^3$) | % |
| medium (100-1,000) | 46 |
| big (1,000-10,000) | 32.76 |
| picoplankton (<10) | 13.73 |
| very big (>10,000) | 5.86 |
| small (10-100) | 1.65 |
| Contrast between 40 – 160 ind $L^{-1}$ pre-grazed communities | |
| Size class ($\mu m^3$) | % |
| big (1,000–10,000) | 41.05 |
| medium (100–1,000) | 23.97 |
| picoplankton (<10) | 21.87 |
| very big (>10,000) | 11.11 |
| small (10–100) | 2 |

The size classes medium (100–1,000 $\mu m^3$) and big (1,000–10,000 $\mu m^3$) cells together contributed most to the differentiation of the pre-grazed communities (Table S1) (Simper Analysis; 0 ind $L^{-1}$ vs 40 ind $L^{-1}$ : 97.32%, 0 ind $L^{-1}$ vs 160 ind $L^{-1}$ : 76.76% and 40 ind $L^{-1}$ vs 160 ind $L^{-1}$: 65.02%). In addition, the positive responses of picoplankton (<10$\mu m^3$) to pre-grazing also

contributed more than 10% to the differentiation of the 160 ind $L^{-1}$ pre-grazed community from the others (Simper Analysis; 0 ind $L^{-1}$ vs 160 ind $L^{-1}$: 13.73% and 40 ind $L^{-1}$ vs 160 ind $L^{-1}$: 21.87%). The change in very big cells (>10,000 $\mu m^3$) contributed also more than 11.11% to the differentiation of the two pre-grazed communities (40 ind $L^{-1}$ vs 160 ind $L^{-1}$) (Table S1).

**Table S2**. ANOVA results showing the short-term effects (days 13) of the factors pre- grazing, nutrient addition and the interaction between them on species growth rate. *=p<0.05 45

| Growth rate | Factor | Df | Sum Sq | Mean Sq | F |
|---|---|---|---|---|---|
| *Chaetoceros sp.* | pre-Grazing | 2 | 0.43 | 0.22 | 4.2* |
| | Nutrients | 1 | 0.87 | 0.87 | 16.7* |
| | pre-Grazing×Nutrients | 2 | 0.02 | 0.01 | 0.2 |
| *Chaetoceros danicus* | pre-Grazing | 2 | 0.19 | 0.10 | 3.9 |
| | Nutrients | 1 | 0.54 | 0.54 | 21.6* |
| | pre-Grazing×Nutrients | 2 | 0.18 | 0.09 | 3.6 |
| *Pseudonitzschia sp.* | pre-Grazing | 2 | 0.24 | 0.12 | 13.8* |
| | Nutrients | 1 | 0.00 | 0.00 | 0.2 |
| | pre-Grazing×Nutrients | 2 | 0.07 | 0.03 | 4* |
| *Guinardia delicatula* | pre-Grazing | 1 | 0.26 | 0.26 | 3.8 |
| | Nutrients | 1 | 0.18 | 0.18 | 2.6 |
| | pre-Grazing×Nutrients | 1 | 0.03 | 0.03 | 0.5 |
| *Skeletonema marinoi* | pre-Grazing | 2 | 0.82 | 0.41 | 8.8* |
| | Nutrients | 1 | 0.60 | 0.60 | 12.9* |
| | pre-Grazing×Nutrients | 2 | 1.00 | 0.50 | 10.6* |
| *Thalassiosira sp.1* | pre-Grazing | 1 | 0.07 | 0.07 | 0.3 |
| | Nutrients | 1 | 0.45 | 0.45 | 1.5 |
| | pre-Grazing×Nutrients | 1 | 0.02 | 0.02 | 0.1 |
| *Thalassiosira sp.2* | pre-Grazing | 2 | 0.99 | 0.49 | 1.7 |
| | Nutrients | 1 | 0.21 | 0.21 | 0.7 |
| | pre-Grazing×Nutrients | 2 | 0.49 | 0.25 | 0.8 |
| *Dactyliosolen fragilissimus* | pre-Grazing | 2 | 1.53 | 0.77 | 3.3 |
| | Nutrients | 1 | 0.34 | 0.34 | 1.4 |
| | pre-Grazing×Nutrients | 2 | 0.39 | 0.19 | 0.8 |
| *Teleaulax sp.* | pre-Grazing | 2 | 0.17 | 0.09 | 0.4 |
| | Nutrients | 1 | 1.02 | 1.02 | 4.5 |

| | | | | | |
|---|---|---|---|---|---|
| | pre-Grazing×Nutrients | 2 | 0.25 | 0.12 | 0.6 |
| *Atheya sp.* | pre-Grazing | 2 | 1.52 | 0.76 | 18.6* |
| | Nutrients | 1 | 0.70 | 0.70 | 17* |
| | pre-Grazing×Nutrients | 2 | 0.15 | 0.08 | 1.9 |

Regarding the major species in these experimental communities, pre-grazing positively affected the growth rates of the species *Chaetoceros danicus*, *Skeletonema marinoi* (highest growth rate at 40 ind $L^{-1}$ -not present at 160 ind $L^{-1}$ pre-grazed treatments) and *Atheya septentrionalis* (highest growth rate at 160 ind $L^{-1}$ pre-grazed treatments) (Figure S7, Table S2). The pre-grazing effect was in contrast negative for the growth rates of *Pseudonitzschia sp.* (highest growth rate at 0 ind $L^{-1}$ pre-grazed treatments). *Chaetoceros* sp. showed a different response to the treatments with the lowest growth rate observed at 40 ind $L^{-1}$ and highest growth rate at 0 ind $L^{-1}$ pre-grazed treatments (Figure S7, Table S2). Nutrients affected significantly both *Chaetoceros* species, *S. marinoi.*, and *A septentrionalis* while for *Pseudonitzschia sp.* and *S. marinoi* (Figure S7, Table S2) the combination of the factors was found significant. *Pseudonitschia sp.* showed an additive negative effect from pre-grazing and nutrient addition while *S. marinoi* pre-grazing effect was positive for the nutrient enriched treatments (N) but negative for the nutrient control treatments (C) (Figure S7, Table S2).
